# Nuclear Magnetic Resonance Spectroscopy Metabolomics in Idiopathic Intracranial Hypertension to Identify Markers of Disease and Headache

**DOI:** 10.1212/WNL.0000000000201007

**Published:** 2022-10-18

**Authors:** Olivia Grech, Senali Y. Seneviratne, Zerin Alimajstorovic, Andreas Yiangou, James L. Mitchell, Thomas B. Smith, Susan P. Mollan, Gareth G. Lavery, Christian Ludwig, Alexandra J. Sinclair

**Affiliations:** Metabolic Neurology (O.G., S.Y.S., Z.A., A.Y., J.L.M., A.J.S.), Institute of Metabolism and Systems Research, College of Medical and Dental Sciences, University of Birmingham; Department of Neurology (A.Y., J.L.M., A.J.S.), University Hospitals Birmingham NHS Foundation Trust; Department of Surgery (T.B.S.), Addenbrooke's Hospital, The University of Cambridge; Birmingham Neuro-Ophthalmology (S.P.M), Queen Elizabeth Hospital, University Hospitals Birmingham; Institute of Metabolism and Systems Research (G.G.L., C.L.), College of Medical and Dental Sciences, University of Birmingham; and Department of Biosciences (G.G.L.), School of Science and Technology, Nottingham Trent University, Clifton Campus, UK.

## Abstract

**Background and Objective:**

We evaluated the metabolomic profile in the CSF, serum, and urine of participants with idiopathic intracranial hypertension (IIH) compared with that in controls and measured changes in metabolism associated with clinical markers of disease activity and treatment.

**Methods:**

A case-control study compared women aged 18–55 years with active IIH (Friedman diagnostic criteria) with a sex-matched, age-matched, and body mass index–matched control group. IIH participants were identified from neurology and ophthalmology clinics from National Health Service hospitals and underwent a prospective intervention to induce disease remission through weight loss with reevaluation at 12 months. Clinical assessments included lumbar puncture, headache, papilledema, and visual measurements. Spectra of the CSF, serum, and urine metabolites were acquired using proton nuclear magnetic resonance spectroscopy.

**Results:**

Urea was lower in IIH participants (CSF, controls median ± IQR 0.196 ± 0.008, IIH 0.058 ± 0.059, *p* < 0.001; urine, controls 5971.370 ± 3021.831, IIH 4691.363 ± 1955.774, *p* = 0.009), correlated with ICP (urine *p* = 0.019) and headache severity (CSF *p* = 0.031), and increased by 12 months (CSF 12 months; 0.175 ± 0.043, *p* = 0.004, urine; 5210.874 ± 1825.302, *p* = 0.043). The lactate:pyruvate ratio was increased in IIH participants compared with that in controls (CSF, controls 49.739 ± 19.523, IIH 113.114 ± 117.298, *p* = 0.023; serum, controls 38.187 ± 13.392, IIH 54.547 ± 18.471, *p* = 0.004) and decreased at 12 months (CSF, 113.114 ± 117.298, *p* < 0.001). Baseline acetate was higher in IIH participants (CSF, controls 0.128 ± 0.041, IIH 0.192 ± 0.151, *p* = 0.008), correlated with headache severity (*p* = 0.030) and headache disability (*p* = 0.003), and was reduced at 12 months (0.160 ± 0.060, *p* = 0.007). Ketones, 3-hydroxybutyrate and acetoacetate, were altered in the CSF at baseline in IIH participants (3-hydroxybutyrate, controls 0.074 ± 0.063, IIH 0.049 ± 0.055, *p* = 0.019; acetoacetate, controls 0.013 ± 0.007, IIH 0.017 ± 0.010, *p* = 0.013) and normalized at 12 months (0.112 ± 0.114, *p* = 0.019, 0.029 ± 0.017, *p* = 0.015, respectively).

**Discussion:**

We observed metabolic disturbances that are evident in the CSF, serum, and urine of IIH participants, suggesting global metabolic dysregulation. Altered ketone body metabolites normalized after therapeutic weight loss. CSF:serum urea ratio was altered, which may influence ICP dynamics and headache. Elevated CSF acetate, known to stimulate trigeminal sensitization, was associated with headache morbidity. These alterations of metabolic pathways specific to IIH provide biological insight and warrant mechanistic evaluation.

**Trial Registration Information:**

Registered on ClinicalTrials.gov, NCT02124486 (submitted April 22, 2014) and NCT02017444 (submitted December 16, 2013).

Idiopathic intracranial hypertension (IIH) is characterized by raised intracranial pressure (ICP), which causes papilledema and a risk of permanent visual loss in addition to chronic headaches, which significantly reduces the quality of life.^1-4^ IIH is becoming more common (indicated by a 350% increased incidence within a decade^5^) and is related to the escalation in worldwide obesity rates.^6,7^ Disease modification can be achieved through weight loss.^8,9^ The underlying cause remains unknown, and this lack of knowledge hinders advances in IIH.

It is well established that IIH occurs almost exclusively (>90%) in women with obesity in association with recent weight gain and truncal adiposity.^9-11^ Knowledge of the disease is, however, advancing, and IIH is no longer considered to be exclusively a central nervous system disease, with mounting evidence indicating systemic metabolic perturbation in excess to that driven by obesity.^11-13^ Patients with IIH are more insulin resistant in the context of hyperleptinemia and adipocyte leptin hypersecretion.^11^ In addition, omental and subcutaneous IIH adipose demonstrates a unique depot-specific lipogenic profile, with adipose tissue transcriptionally primed for increased calorie storage.^11^ Systemic hormonal dysregulation has been noted in IIH with a distinct profile of androgen excess identified.^12^ In addition, metabolic targeting by blocking the cortisol-generating enzyme 11β hydroxysteroid dehydrogenase type 1 has shown therapeutic potential in IIH.^14^ Patients with IIH also have a doubled risk of cardiovascular disease when compared with those with obesity alone.^6^

Defining the etiology of IIH and identifying biomarkers to guide diagnosis were deemed top priorities for research, by both health care professionals and patients with IIH in a priority setting partnership.^15^ Metabolites have not been previously quantified in IIH in comparison with controls matched for obesity. We suggest that investigation of the metabolic pathways involved in IIH may shed light on disease pathogenesis and have relevance for developing targeted therapeutics.

This study used an untargeted metabolomic method to identify quantitative differences in metabolites in the CSF, serum, and urine of active IIH participants using proton nuclear magnetic resonance spectroscopy, in comparison with an age-matched, sex-matched, and body mass index (BMI)-matched control group. Subsequently, we sought to identify the relationship between metabolites and clinical measurements and finally to determine alterations in metabolites at 12 months after disease treatment.

## Methods

### Study Design

A case-controlled study compared metabolite concentrations between control and IIH participants at baseline. A subgroup of IIH participants subsequently underwent a prospective intervention study, which evaluated methods of weight loss to achieve disease remission over 12 months. The clinical trial protocol and clinical results of the weight loss intervention have been reported elsewhere.^8,16^

### Standard Protocol Approvals, Registrations, and Patient Consents

The study was approved by the National Research Ethics Committees from the West Midlands–The Black Country (14/WM/0011) and York and Humber-Leeds West approved (13/YH/0366)]. In accordance with the Declaration of Helsinki, all participants gave written informed consent to participate in the study. The detailed trial methodology, protocol, and statistical analysis plan have been published.^14,16,17^ This substudy is reporting on registered trials NCT02124486 and NCT02017444 and was an exploratory objective of the IIH:WT trial. Written informed consent was obtained from all participants in the study.

### Participants

#### Inclusion and Exclusion Criteria

IIH participants were identified from neurology and ophthalmology clinics from 7 United Kingdom National Health Service hospitals between March 2014 and May 2017. In brief, the inclusion criteria for IIH participants featured the following: female sex, aged between 18 and 55 years, BMI ≥35 kg/m^2^, clinical diagnosis of active IIH meeting the Friedman diagnostic criteria^18^ (ICP ≥25 cmCSF >2 months duration and active papilledema (Frisen grade >1 in at least 1 eye)), normal brain imaging (including magnetic resonance venography or CT with venography), apart from radiologic signs of raised ICP, and ability to give informed consent.

In brief, exclusion criteria included the following: aged younger than 18 years or older than 55 years, pregnancy, having undergone optic nerve sheath fenestration, significant comorbidities including known endocrinopathies, receiving hormone manipulating medication, the inability to perform a visible field reliably, whether there was a secondary cause of raised ICP, or whether the IIH had gone into remission (absence of papilledema). Control participants were recruited through advertising on social media and included women aged between 18 and 55 years with obesity (BMI ≥35 kg/m^2^) and with analogous exclusion criteria to the IIH participants.

#### Assessments

At baseline, all participants underwent a detailed medical history and examination. A headache diary recorded monthly headache days (days per month) and severity (numerical rating of scale 0–10 with 10 denoting maximum pain). Headache disability was evaluated using the Headache Impact Test (HIT)-6 questionnaire. BMI was calculated from weight and height and using the following formula: BMI = (weight (kg)/height (m)^2^). A lumbar puncture (LP) was conducted in all participants in the lateral decubitus with knees bent at a 90° angle or more and lumbar puncture opening pressure (LPOP) recorded before the CSF was collected (up to 15 mL). Visual assessments included automated perimetry with the Humphrey Visual Field (HVF) Analyzer (Carl Zeiss Ophthalmic Systems, Inc) using a 24-2 Swedish Interactive Threshold Algorithm (SITA) standard test pattern. The worse eye was identified for each patient by defining the eye with the more severe perimetric mean deviation (PMD). The optic nerve head swelling was measured using spectral domain optical coherence tomography (OCT; Spectralis, Heidelberg Engineering) to evaluate the average peripapillary retinal nerve fiber layer (RNFL) thickness.

### Sample Collection and Preparation

Participants were fasted overnight (from midnight) before the CSF and blood serum sample collection at the study visit. Samples were transported on ice and centrifuged within 30 minutes (10 minutes 1500*g* for blood, 800*g* for the CSF) at 4°C. Twenty-four-hour urine samples were also provided by participants on attendance to the research facility. All samples were stored at −80°C and analyzed after a maximum of 1 freeze-thaw cycle.

### Metabolite Extraction

For serum and CSF samples, metabolites were extracted before processing. In brief, methanol (−80°C) was added to all samples to quench metabolism. Samples were then incubated on wet ice, and chloroform was added to aid the separation of nonpolar metabolites. This mixture was vortexed and centrifuged at 4°C to enable separation of polar metabolites. Two mL of the polar fraction was transferred to a new tube and dried. The polar phase was then reconstituted in sodium phosphate buffer (100 mM sodium phosphate, 500 μM 4,4-dimethyl-4-silapentane-1-sulfonic acid and 2 mM Imidazole in 100% Deuterium oxide).

Urine samples were manually pH adjusted (target pH, 7.0) and mixed with concentrated NMR buffer, yielding an endpoint phosphate buffer concentration of 100 mM, 10% D20, 0.5 mM Sodium trimethylsilylpropanesulfonate (DSS), and 2 mM imidazole.

### Acquisition and Preprocessing of ^1^H-NMR Spectra

One-dimensional ^1^H-NMR spectra were obtained from all samples using a 600 MHz Bruker Avance III spectrometer with a 1.7-mm z-PFG TCI Cryoprobe at 300K. Solvent suppression was achieved using the NOESY presaturation pulse sequence. Spectral width was set to 12.2 ppm, and 16,384 complex data points were acquired with a 4.0s interscan relaxation delay. All NMR spectra were processed using the MetaboLab software.^19^ Free induction decays were apodized using an exponential line broadening of 0.3 Hz and zero-filled to 131,072 real data points before Fourier transformation. The DSS internal standard signal in each spectrum was referenced to 0.0 ppm, followed by manual phase correction and batch baseline correction using a spline baseline before export int Bruker format. Chenomx NMR Suite version 8.3 (Chenomx Inc, Alberta, Canada) was used to conducted comprehensive, untargeted metabolite annotation and quantification.

### Normalization of Data

Serum datasets were normalized before statistical analysis using probabilistic quotient normalization (PQN)^20^ on MetaboAnalyst 4.0^21^ to remove variation due to dilution factors. Normalization was not necessary for CSF because it is subject to tight homeostatic regulation and is unlikely to vary in concentration between individuals.^22^

### Statistical Analyses

This was a prospective evaluation, and we reported the primary analysis of these data. All univariate statistical analyses were performed using SPSS Statistics version 25.0 (IBM Corp, Armonk, NY, USA). No pretreatment of data was performed before these analyses.^23^ Figures were produced using GraphPad Prism version 8.0 (GraphPad Software Inc, San Diego, CA, USA).

The normality of data was assessed using quantile-quantile plots and the Shapiro-Wilk test. Distributions that were normally distributed were compared using parametric tests (t tests), whereas nonparametric tests (the Mann-Whitney test, Spearman rank correlation test, and Wilcoxon signed rank test for baseline and 12 months paired data) were used in the case of distributions that were not normally distributed. Continuous clinical characteristic data were reported as mean and standard deviation (SD), and metabolites were reported as median and interquartile range (IQR) and analyzed as nonparametric data. Where data points were missing, data were not imputed. Statistical significance was defined as *p* < 0.05. Because the study did not aim to delineate diagnostic biomarkers, but instead to understand metabolic pathways, deriving conclusions from multiple pathways and metabolites, data were not corrected for multiple testing.

### Data Availability

Anonymized individual participant data may be made available along with the trial protocol. Proposals should be made to the corresponding author and will be reviewed by the Birmingham Clinical Trials Unit Data Sharing Committee in discussion with the Chief Investigator. A formal Data Sharing Agreement may be required between respective organizations once release of the data is approved and before data can be released.

## Results

### Metabolite Concentrations in IIH Participants Relative to Controls

All participants were female with a mean ± SD age of 36.60 ± 8.47 years in controls (n = 20) and 33.01 ± 7.11 years in IIH participants (n = 84) (Table 1). BMI-matched controls had a BMI of 43.74 ± 4.96 m^2^/kg, whereas IIH participants had a BMI of 42.24 ± 7.90 m^2^/kg. As expected, controls had a significantly lower LPOP pressure (23.74 ± 3.81 cmCSF controls vs 34.03 ± 5.52 cmCSF, *p* < 0.001, Table 1).

**Table 1:**
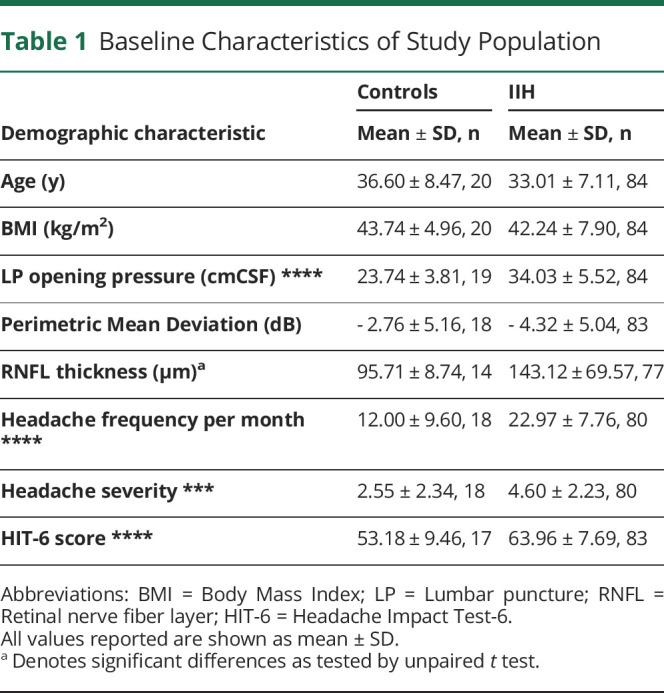
Baseline Characteristics of Study Population

A distinct metabolic profile was identified in IIH participants at baseline in comparison with controls featuring 23 CSF metabolites, 12 serum metabolites, and 9 urine metabolites (Table 2), which significantly differed in concentration between IIH participants and controls. We have discussed in detail the differential metabolites that were most consistently associated with clinical measures and normalized after 12 months.

**Table 2:**
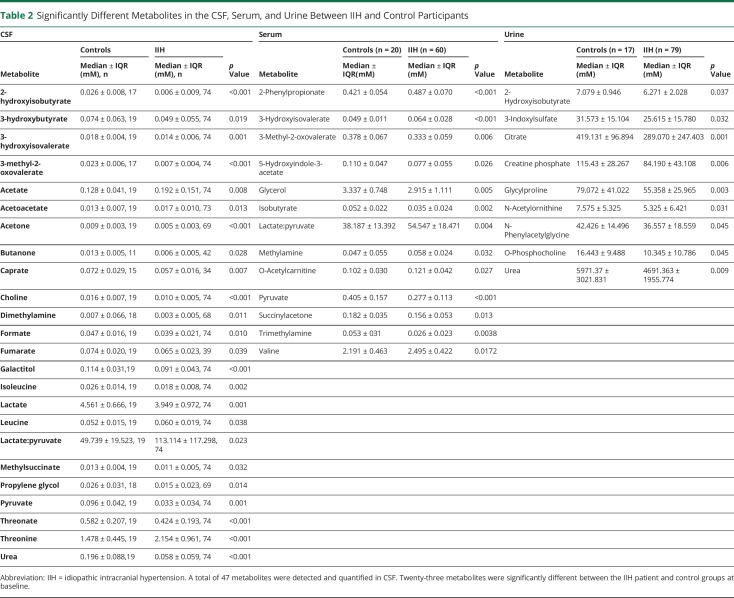
Significantly Different Metabolites in the CSF, Serum, and Urine Between IIH and Control Participants

**Figure 1:**
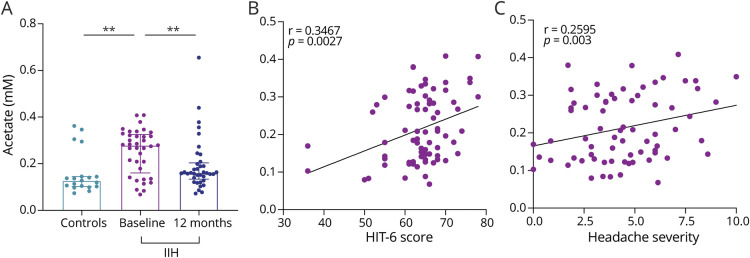
Changes in Acetate Concentration and Its Association With Headache in IIH Participants (A) Acetate concentration is significantly higher in CSF in IIH than controls and reduced at 12 months. (B) Acetate is associated with headache disability (HIT-6) scores at baseline in IIH. (C) Acetate is also associated with headache severity at baseline in IIH. IIH = idiopathic intracranial hypertension.

**Figure 2:**
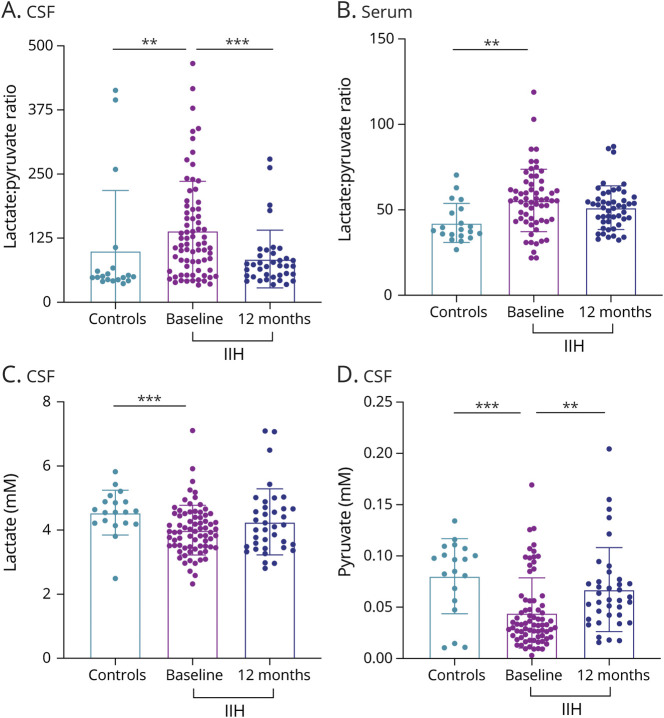
Lactate:Pyruvate Measurements in IIH in Comparison With Controls and at 12 Months (A) Lactate:pyruvate ratio in the CSF in controls in comparison with IIH participants at baseline and 12 months. (B) Lactate:pyruvate ratio in serum in controls and IIH participants at baseline. (C) Lactate ratio in the CSF in controls and IIH participants at baseline and 12 months. (D) Pyruvate ratio in the CSF in controls and IIH participants at baseline and 12 months. IIH = idiopathic intracranial hypertension.

**Figure 3:**
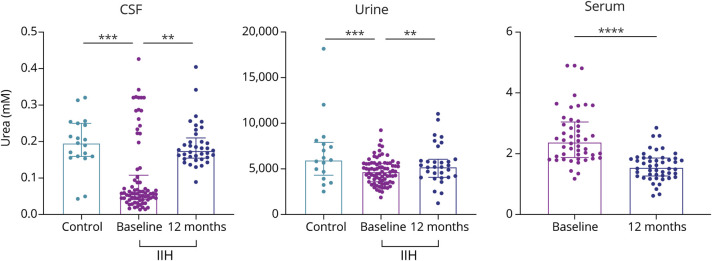
Urea Concentrations in the CSF, Serum, and Urine and Their Correlations With Clinical Measurements (A) CSF and (B) Urine urea in controls and IIH participants at baseline and 12 months. (C) Serum urea in IIH at baseline and 12 months. IIH = idiopathic intracranial hypertension.

Within the CSF analysis, we identified that acetate was significantly higher in IIH participants than controls (0.128 ± 0.041 control vs 0.192 ± 0.151, *p* = 0.008, Figure 1A). Moreover, the lactate:pyruvate ratio, a marker of mitochondrial dysfunction, was significantly higher in IIH participants than in controls (49.739 ± 19.523 control vs IIH 113.114 ± 117.298, *p* = 0.023, Figure 2A). Individual lactate and pyruvate measurements, products of glycolysis, were significantly lower in IIH participants compared with those in controls (lactate 4.561 ± 0.666 control vs 3.949 ± 0.972 IIH, *p* = 0.001, Figure 2C, pyruvate 0.096 ± 0.042 control vs 0.033 ± 0.034 IIH, *p* = 0.001, Figure 2D). There was no difference in the lactate:pyruvate ratio or lactate concentrations in those taking acetazolamide compared with those who were not at baseline (medication use; eTable 1 in the Supplement, links.lww.com/WNL/C289). Fumarate, a citric acid cycle metabolite, was also lower in IIH participants in comparison with that in controls (0.074 ± 0.020 controls vs 0.065 ± 0.023 IIH, *p* = 0.039). Urea, an osmolar metabolite, was significantly lower in IIH participants than in controls (0.196 ± 0.088 controls vs 0.058 ± 0.059 IIH, *p* < 0.001, Figure 3A). Ketones 3-hydroxybutyrate and acetoacetate were also altered in IIH participants in comparison with that in controls (3-hydroxybutyrate 0.074 ± 0.063 controls vs 0.049 ± 0.055 IIH, *p* = 0.019, acetoacetate 0.013 ± 0.007 controls vs 0.017 ± 0.010, *p* = 0.013, Table 2).

Although there were fewer differential metabolites in the serum (12 metabolites, Table 2), there were similarities to the differential metabolites in the CSF, with the lactate:pyruvate ratio being significantly higher in the serum of IIH participants in comparison with that in controls (38.187 ± 13.392 control vs 54.547 ± 18.471 IIH, *p* = 0.004, Figure 2B). Moreover, individual pyruvate measurements were also significantly lower in IIH participants compared with that in controls (controls 0.405 ± 0.157 vs IIH 0.277 ± 0.113, *p* < 0.001).

Of note, of the 9 differential urine metabolites (Table 2), urea was significantly lower in IIH participants than in controls (controls 5071.37 ± 3021.83 vs 4691.363 ± 1955.774 IIH *p* = 0.009, Figure 3B), a finding also observed in the CSF. Citrate, a citric acid cycle metabolite, is significantly lower in IIH participants in comparison with that in controls (419.131 ± 96.894 controls vs 289.070 ± 247.403 IIH, *p* = 0.001).

### Metabolite Changes at the 12-Month Follow-up

We sought to explore any changes in metabolite concentrations by prospectively reevaluating IIH participants at 12 months after a therapeutic weight loss intervention. Over the follow-up period, we noted a mean ± SD reduction in BMI of 6.18 ± 7.68 kg/m^2^ and a reduction in LPOP of 10.3 ± 12.55 cmCSF (eTable 2 in the Supplement, links.lww.com/WNL/C289).

Of the 23 CSF metabolites that were significantly different between controls and IIH participants at baseline, 13 changed significantly at 12 months in IIH participants (Table 3). The elevated CSF acetate noted at baseline was reduced at 12 months (0.192 ± 0.151 baseline vs 0.160 ± 0.060 12 months, *p* = 0.007, Figure 1A). Of importance, the lactate:pyruvate ratio, which was higher in IIH participants than in controls at baseline, was reduced at the 12-month follow-up and no longer significantly different to controls (baseline 113.114 ± 117.298 vs 12 months 70.776 ± 39.050 *p* = 0.004, Figure 2A). The reduced CSF urea identified at baseline significantly increased at 12 months (0.058 ± 0.059 baseline vs 0.175 ± 0.043 12 months, *p* = 0.004, Figure 3A). Ketones acetoacetate and 3-hydroxybutyrate also normalized at the 12-month follow-up (acetoacetate 0.017 ± 0.031 baseline vs 0.029 ± 0.017, *p* = 0.015, 3-hydroxybutyrate 0.074 ± 0.063 baseline vs 0.112 ± 0.114 12 months, *p* = 0.019).

**Table 3:**
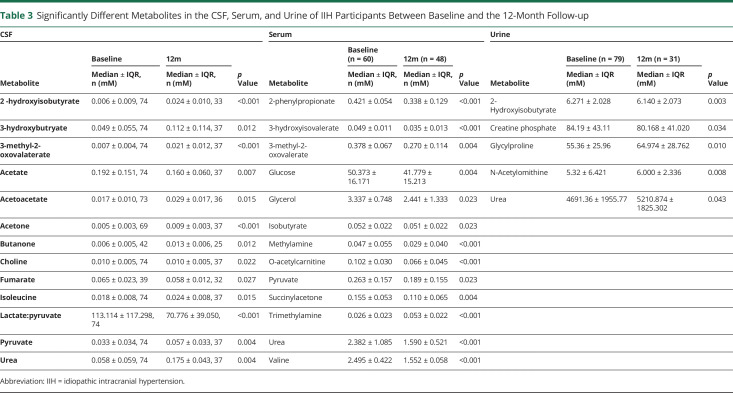
Significantly Different Metabolites in the CSF, Serum, and Urine of IIH Participants Between Baseline and the 12-Month Follow-up

In serum, of the 12 differential metabolites identified in IIH participants at baseline, 11 were significantly altered at 12 months (Table 3). Of interest, urea was significantly lower at 12 months in serum than at baseline (baseline 2.382 ± 1.085 vs 12 months 1.552 ± 0.576, *p* < 0.001, 3C). The CSF:serum urea ratio was significantly increased at 12 months, reflecting a decrease in serum urea and increase in CSF urea at 12 months (baseline 0.025 ± 0.083 vs 12 months 0.115 ± 0.058, *p* < 0.001). Pyruvate was also significantly lower at 12 months than at baseline (baseline 0.263 ± 0.157 vs 12 months 0.189 ± 0.155, *p* = 0.022).

Of the 9 differential metabolites identified in urine, 5 were significantly changed at 12 months in IIH participants (Table 3), 2 of which were increased to concentrations akin to controls, including glycylproline (baseline 55.36 ± 25.96 vs 12 months 64.974 ± 28.762, *p* = 0.010) and urea (baseline 4691.36 ± 1955.77 vs 12 months 5210.874 ± 1825.302 *p* = 0.043, Figure 3B).

### Disease Remission

Fifteen of 45 patients with matched data had an ICP <25cmCSF at 12 months and therefore were in disease remission. As described, 3-hydroxybutryate, a ketone body that was significantly lower in IIH participants than in controls at baseline and significantly increased at 12 months, demonstrated a greater increase in the CSF of the disease remission group (0.080 ± 0.067 baseline vs 0.163 ± 0.109 12 months *p* = 0.020). Acetoacetate, another ketone body that was differential between IIH participants and controls at baseline and significantly increased at 12 months, exhibited a greater increase in the disease remission group (0.0148 ± 0.007 baseline vs 0.0377 ± 0.020, *p* = 0.004). Serum glucose, which significantly decreased at 12 months, demonstrated a greater reduction in those in disease remission (56.318 ± 19.046 baseline vs 40.989 ± 11.187 12 m *p* = 0.005).

### Associations Between Metabolites and Clinical Measurements

The relationship between the IIH-specific metabolites and clinical features were then evaluated. ICP measured by LP was associated with galactitol in the CSF, pyruvate and 2-hydroxyisobutyrate in the serum, and urea in the urine (eTable 3 in the Supplement, links.lww.com/WNL/C289). ICP was significantly reduced at the 12-month follow-up (eTable 2 in the Supplement, links.lww.com/WNL/C289), and the change in ICP was associated with change in butanone in the CSF and o-phosphocholine and N-phenylacetylglycine in the urine (eTable 4 in the Supplement, links.lww.com/WNL/C289).

Raised ICP in IIH drives papilledema, the hallmark sign of disease activity with subsequent risk of visual loss. Papilledema was evaluated using the OCT RNFL, and measurements were associated with isobutyrate and O-acetylcarnitine in the serum and 2-hydroxyisobutyrate in the urine (eTable 3 in the Supplement, links.lww.com/WNL/C289). The change in RNFL thickness was associated with pyruvate change in the CSF (eTable 4 in the Supplement, links.lww.com/WNL/C289). Visual function, measured by perimetric mean deviation, was associated at baseline with fumarate, lactate, and threonate in the CSF, 5-hydroxyindole-3-acetate in the serum, and citrate in the urine (eTable 3 in the Supplement, links.lww.com/WNL/C289). Changes in the perimetric mean deviation were associated with changes in leucine in the CSF in addition to valine, trimethylamine, 3-methyl-2-oxovalerate, and urea in the serum (eTable 4 in the Supplement, links.lww.com/WNL/C289).

Headache in IIH is initiated by elevated ICP and causes significantly reduced quality of life, yet the underlying mechanism driving pain is not understood.^3,24^ We explored the relationship between metabolites and headache morbidity. Headache disability, measured by the HIT-6, was associated with 3-hydroxybutyrate and acetate in the CSF at baseline (acetate Figure 1B, eTable 3 in the Supplement, links.lww.com/WNL/C289). Headache severity was associated with acetate (Figure 1C) and urea in the CSF and creatine phosphate in the urine. Monthly headache days were also significantly associated with creatine phosphate in the urine (eTable 3 in the Supplement, links.lww.com/WNL/C289). At the 12-month follow-up, changes in HIT-6 were associated with changes in butanone in the CSF and urea in the urine. Changes in headache severity were associated with changes in pyruvate and were significantly associated with changes in multiple metabolites in the urine including butanone, 2-hydroxyisobutyrate, and creatine phosphate (eTable 4 in the Supplement, links.lww.com/WNL/C289).

Obesity is a typical feature of IIH and has been implicated in disease etiology. We sought to evaluate which metabolites were associated with BMI. At baseline, BMI was significantly associated with multiple metabolites in CSF including 3-methyl-2-oxovalerate, isoleucine, methylsuccinate, propylene glycol, and threonine (eTable 3 in the Supplement, links.lww.com/WNL/C289). In urine, 3-Indoxylsulfate, N-acetylornithine, and N-phenylacetylglycine were also significantly associated with BMI. After 12 months of weight loss intervention, changes in BMI were significantly associated with butanone in the CSF, and importantly, urea, 3-indoxylsulfare, and N-phenylacetylglycine in the urine (eTable 4 in the Supplement, links.lww.com/WNL/C289).

## Discussion

Despite the phenotype of IIH being stereotyped in women with obesity, the etiology has remained elusive. We have used untargeted, quantitative metabolite phenotyping in a large cohort of active IIH participants. We have identified a profile of disease specific metabolites that are associated with clinical measures and markers of disease activity. The results provide biological insights and point toward remodeling of metabolite pathways in IIH. The dominating signals were dysregulation of the lactate:pyruvate ratio, a marker of respiratory chain metabolism, and altered ketone body metabolism. We also noted metabolic changes in the urea CSF:serum ratio and acetate metabolism, which maybe critical in contributing to the severe headache phenotype in IIH.

The lactate:pyruvate ratio was repeatedly altered in IIH and is an established marker of anaerobic metabolism and mitochondrial energy metabolism disorders, such as oxidative phosphorylation disorders and pyruvate dehydrogenase deficiency.^25,26^ We identified an increased ratio at baseline in IIH participants compared with that in controls in both the CSF and serum. Elevated CSF lactate:pyruvate ratio is a feature of numerous conditions of raised ICP including traumatic brain injury,^27^ subarachnoid hemorrhage^28,29^ and hydrocephalus,^30^ suggesting that these metabolic alterations may be a feature of raised ICP. Fumarate, a citric acid cycle intermediate, is also reduced in the CSF of patients with IIH, contributing toward the readout of dysfunctional respiratory metabolism in the brain (eFigure 1, links.lww.com/WNL/C289). Significant reduction in lactate:pyruvate ratio in CSF at 12 months is consistent with findings in hydrocephalus, in which ratios are highest in the setting of elevated ICP and reduced after ventriculoarterial shunt insertion in association with reduced ICP.^30^

**Figure 4:**
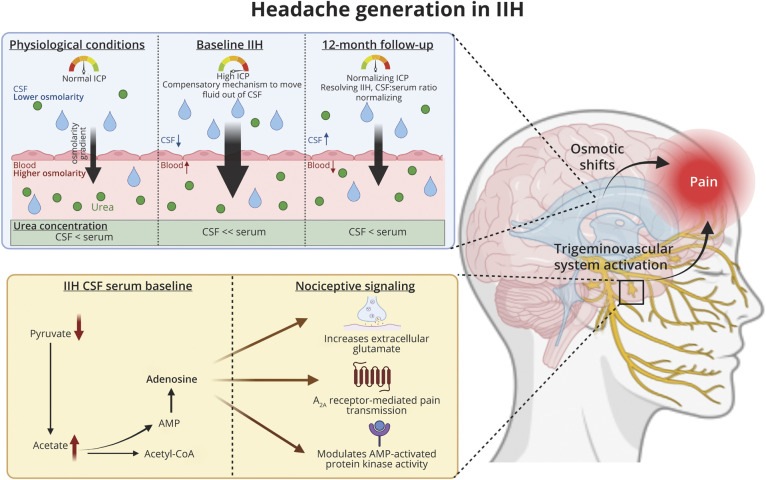
Perturbed Metabolic Pathways in IIH, Which May Contribute Toward Headache Generation Changes in urea concentration in IIH may indicate compensatory adaptive biological mechanisms to regulate fluid movement in the brain. Hypothesized compensatory mechanism in which urea, a hyperosmolar agent, directs the movement of water from low to high concentrations. CSF:serum ratio is significantly increased at 12 months in IIH. Metabolic pathway of acetate production and its products that are implicated in trigeminal sensitizing pathways and may contribute toward headache generation in IIH. IIH = idiopathic intracranial hypertension.

Urea, a hyperosmotic metabolite, was noted to differ in IIH. Urea was reduced in IIH at baseline in the CSF and urine. The urea levels normalized in line with disease resolution and falling ICP at 12 months. In physiologic conditions, CSF urea concentrations are slightly lower than in serum.^31^ We suggest that in IIH, the reduced CSF urea, relative to the serum, may represent a compensatory mechanism that increases the osmotic gradient allowing more fluid to move out of the CSF (Figure 4). After treatment, in which ICP is reduced in IIH, the CSF:serum ratio normalizes. The osmotic properties of urea are used therapeutically with intravenous urea administration being used extensively to reduce brain swelling in a range of conditions,^32^ and in particular was able to significantly reduce ICP in patients with acute brain injury, especially in those with the highest ICP among the cohort (ICP ≥15 mm Hg).^33^ Traumatic brain injury (TBI) models in mice have also demonstrated alterations in the expression of urea transporters, which was believed to be an adaptive modulation for changing urea levels.^34^

Of interest, we also demonstrated a negative relationship between CSF urea and headache severity (with high CSF urea being associated with increased headache severity). These findings suggest that alteration of the CSF:serum urea gradient in IIH may be important in driving headache (Figure 4). The importance of urea and perturbed osmotic gradients is noted in individuals after hemodialysis who frequently experience headaches.^35^

We also identified alterations in acetate, a metabolite that is converted to acetyl CoA for multiple metabolic reactions including ketone body production and the citric acid cycle. Acetate concentrations are significantly higher in IIH at baseline and are associated with headache disability (HIT-6) and headache severity. The conversion of acetate into acetyl CoA through acetyl CoA synthase yields AMP and adenosine, which mediates pain transmission through stimulation of nociceptive nerve terminals through adenosine A2A receptors^36^ and release of pain mediators such as histamine by mast cells.^37^ Acetate is also the main metabolite responsible for headache symptoms in “hangover headache,^38^ and administration is able to increase sensitivity of headache pathways through the trigeminal system in rodent migraine models.^38^ The use of acetate as a buffer for kidney dialysis also led to headaches experienced by patients.^39^ IIH headaches are known to have migraine-like features.^24^ Although we did not measure adenosine, higher concentrations of acetate could also contribute toward trigeminal sensitivity in patients with IIH and may contribute to the etiology of headaches in IIH (Figure 4). Acetate is also significantly reduced at 12 months in IIH participants after disease remission and reduction in headache measurements. Expression of acetyl CoA synthase has been found to be suppressed in leptin deficiency. Leptin resistance has been demonstrated in IIH^11^ and may have a role in modulating the expression of acetyl CoA synthase and its ability to metabolize acetate.

The ketone body acetoacetate is significantly higher in the CSF at baseline in IIH, again indicating an upregulation of ketone formation. Ketogenesis is similarly upregulated during fasting and insulin resistance. Previous IIH studies have identified insulin insensitivity in IIH,^11^ which may lead to an increased acetoacetate production. However, reduced 3-hydroxybutyrate and acetone concentrations in the CSF suggest that either conversion of acetoacetate to these ketone bodies is inhibited or that these ketone bodies are used as an alternative energy source. After 12 months, acetone and 3-hydroxybutyrate are significantly increased in IIH. Increase in these ketone bodies is also exhibited early in patients after bariatric surgery in serum and was believed to be due to an increased lipolytic activity.^40^ Because IIH participants have significant weight loss at 12 months, ketone body formation may continue because fat loss is achieved and lipolysis continues. Moreover, in a subanalysis, we noted that acetoacetate was significantly different between the diet and bariatric surgery arm, with acetoacetate being significantly higher in the surgery arm at 12 months (*p* < 0.05). It is possible that changes in ketones at 12 months may be driven by weight loss or the metabolic changes after bariatric surgery. We also noted a reduction in serum glucose at 12 months, which may reflect changes attributed to weight loss. However, because this was not associated with any changes in BMI, we cannot be certain.

The study includes some potential confounds, one of which is the smaller number of control participants in comparison with IIH participants. Participants were matched by age, sex, and BMI, which limited the number of controls eligible for inclusion. However, obtaining detailed phenotyping and CSF collections from healthy BMI-matched controls is challenging due to ethical considerations and participant acceptability. Despite this, we presented the largest group of controls matched for age, sex, and BMI of any IIH studies. Because this study was limited to including women only, the results may not be generalizable to children and men with IIH. We found that not all clinical markers (including papilledema) were correlated with metabolites. This may relate to the severity of disease in participants; therefore performing this analysis in a more severe disease cohort would be of future interest. Another strength of our study is that we were able to review metabolite profiles at the 12-month follow-up. However, this led to some missing data, decreasing the amount of paired data; this was due to the patients' decision to not have a sample taken at that time point. We acknowledge that a small number of patients were on medications and cannot exclude that this may have affected the metabolite profile; however, there were no significant changes noted to result from medications, for example, acetazolamide. Therefore, it is unlikely that the medications meaningfully alter the inference of our data.

In this quantitative metabolomics study of IIH, we report a distinct metabolomic profile in the serum, CSF, and urine of IIH participants in comparison with sex-matched, age-matched, and BMI-matched control group. The results provide a preliminary mechanistic insight into the pathogenesis of IIH, implicating metabolic dysfunction that manifests in an increased lactate:pyruvate ratio and altered ketone body concentrations. We have also identified metabolic perturbations that are implicated in the mechanisms driving the severe chronic headaches in IIH. Acetate is elevated in IIH and known to stimulate trigeminal sensitization. Alterations in the urea CSF:serum ratio, a contributor to osmotic gradient and fluid shifts, was noted in IIH and was also associated with headache pain.

These findings extend our knowledge of IIH etiology and provide a roadmap for future mechanistic studies. Further work is required to validating our findings and to establish which metabolites may be the most clinically useful as biomarkers of disease diagnosis, progression, and outcome. This may include instigation of the role of these key metabolites in IIH pathogenesis in animal and cell models.

## Glossary

BMI: Body Mass Index
IIH: idiopathic intracranial hypertension
LP: Lumbar puncture
RNFL: Retinal nerve fiber layer
HIT-6: Headache Impact Test-6
